# Aryl hydrocarbon receptor pathway activation enhances gastric cancer cell invasiveness likely through a c-Jun-dependent induction of matrix metalloproteinase-9

**DOI:** 10.1186/1471-2121-10-27

**Published:** 2009-04-16

**Authors:** Tie-Li Peng, Jie Chen, Wei Mao, Xin Song, Min-Hu Chen

**Affiliations:** 1 Department of Gastroenterology, the First Affiliated Hospital of Sun Yat-Sen University, Guangzhou, PR China

## Abstract

**Background:**

Abberant aryl hydrocarbon receptor (AhR) expression and AhR pathway activation are involved in gastric carcinogenesis. However, the relationship between AhR pathway activation and gastric cancer progression is still unclear. In present study, we used 2,3,7,8-tetrachlorodibenzo-para-dioxin (TCDD), a classic and most potent ligand of AhR, to activate AhR pathway and investigated the effect of AhR pathway activation on human gastric cancer AGS cell invasion and explored the corresponding mechanism.

**Results:**

To determine whether AhR pathway can be activated in AGS cells, we examined the expression of CYP1A1, a classic target gene of AhR pathway, following TCDD exposure. RT-PCR and western blot analysis showed that both CYP1A1 mRNA and protein expression were increased in a dose-dependent manner following TCDD treatment and AhR antagonist resveratrol (RSV) could reverse this TCDD-induced CYP1A1 expression. To determine whether TCDD treatment of AGS cells results in an induction of MMP-9 expression, we detected MMP-9 mRNA using RT-PCR and detected MMP-9 enzymatic activity using gelatin zymography. The results showed that both MMP-9 mRNA expression and enzymatic activity were gradually increased with the concentration increase of TCDD in media and these changes could be reversed by RSV treatment in a dose-dependent manner. To examine whether AhR activation-induced MMP-9 expression and activity in AGS cells results in increased migration and invasion, we performed wound healing migration assay and transwell migration and invasion assay. After TCDD treatment, the migration distance and the migration and invasion abilities of AGS cells were increased with a dose-dependent manner. To demonstrate AhR activation-induced MMP-9 expression is mediated by c-Jun, siRNA transfection was performed to silence c-Jun mRNA in AGS cells. The results showed that MMP-9 mRNA expression and activity in untreated control AGS cells were very weak; After TCDD (10 nmol/L) treatment, MMP-9 mRNA expression and activity were significant increased; This TCDD-induced MMP-9 expression and activity increase could be abolished by c-Jun siRNA transfection.

**Conclusion:**

AhR pathway activation enhances gastric cancer cell invasiveness likely through a c-Jun-dependent induction of MMP-9. Our results provide insight into the mechanism and function of the AhR pathway and its impact on gastric cancer progression.

## Background

Aryl hydrocarbon receptor (AhR) is a ligand-activated transcription factor of the basic helix-loop-helix/Per-Arnt-Sim family. In the absence of ligand, AhR is present in the cytosol in the form of a complex with two chaperone Hsp90s, a smal protein (p23), and an immunophilin-like protein (XAP2) [1,2]. Upon ligand such as 2,3,7,8-tetrachlorodibenzo-para-dioxin (TCDD, the most potent and classical exogenous AhR ligand) binding, the chaperon proteins dissociate and AhR translocate into the nucleus to form a heterodimer with its partner molecule aryl hydrocarbon receptor nuclear translocator (ARNT) [3,4]. This heterodimer binds to the specific DNA region termed dioxin response element (DRE), which has a core sequence of 5'-TNGCGTG-3', and thereby activates a battery of genes expression [5-7].

Historically, studies of AhR pathway have focused on the transcriptional regulation of genes encoding xenobiotic metabolizing enzymes such as cytochrome P450 enzymes [8]. Recent studies demonstrated a close relationship between AhR and mammary gland tumorigenesis [7,9]. AhR gene polymorphisms have been linked to an increased risk of lung and breast cancers [10,11]. Increased expression of AhR has been reported in lung, breast, and pancreatic cancers in humans [7,12,13]. Studies also suggest that constitutively active AhR may promote hepatocarcinogenesis in mice [14]. These data indicated a close relationship between AhR and tumorigenesis. However, the relationship between AhR and tumor progression is not clear.

Tumor cells invasion and metastasis is a complicated process among which degradation of extracellular matrix (ECM) and basement membrane is a crucial step. Tumor invasion and metastasis relies on the expression of matrix metalloproteinases (MMPs) to destroy the ECM and basement membrane to allow cell migration. MMPs are a group of zinc dependent metallopeptidases [15-17]. Matrix metalloproteinase-9 (MMP-9) is one of the type IV collagenase/gelatinases, which degrade basement membrane collagens and gelatins [16]. MMP-9 is widely associated with tumor invasion and metastasis [17]. The synthesis of MMP-9 is regulated by several growth factors, cytokines and hormones [16,18]. Recent study linked TCDD-associated lesions with aberrant matrix metabolism [8]. Microarray data demonstrate that TCDD/AhR alter expression of genes involve in matrix metabolism and deposition [8]. Villano et al [19] and Haque et al [18] reported that AhR agonist TCDD could induce MMP-9 expression in huamn melanoma cells and prostate cancer cells. These studies suggest that the MMP-9 expression may be a common endpoint for activation of the AhR pathway [8,19].

Gastric cancer is the fourth most common malignancy and the second most frequent cause of cancer-related death in the world [20]. Gastric cancer cells invasion and metastasis often lead to a poor prognosis. Several studies linked AhR pathway activation to gastric carcinogenesis. Chen et al found increased expression of AhR in two human gastric cancer cell lines (RF1 and RF48) by microarray analysis [21]. Ma et al reported that concurrent expression of AhR and CYP1A1 is correlated with gastric cancer development [22]. Andersson et al found that constitutively activated AhR could induce stomach tumors in a transgenic mouse model [23]. In another of our studies, we found that AhR expression and nuclear translocation were significant higher in gastric cancer than in premalignant lesions and normal gastric mucosa [24]. However, the relationship between AhR pathway activation and gastric cancer invasion and metastasis is still not clear. Therefore, we investigated the effect of AhR pathway activation on human gastric cancer cells. Our data presented here demonstrate that AhR pathway can be activated in gastric cancer AGS cells and AhR pathway activation in AGS cells induces MMP-9 expression and enhances AGS cells migration and invasion activity. Furthermore, our data show that this AhR activation-induced MMP-9 expression is mediated by c-Jun.

## Results and discussion

### AhR pathway activation in AGS cells

**Figure 1:**
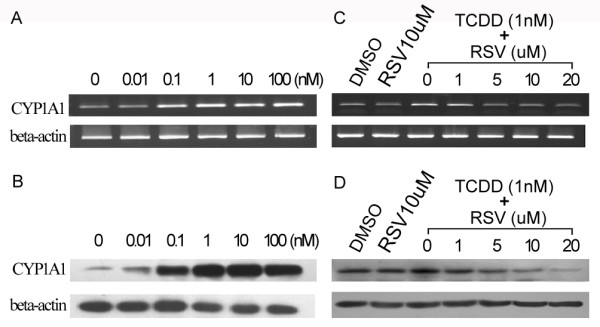
**AhR agonist TCDD and AhR antagonist RSV regulated CYP1A11 expression in AGS cells**. After different concentrations (as shown above) of TCDD treatment for 24 hours, (A) RT-PCR analysis of CYP1A1 mRNA expression in a concentration-response. (B) Western blot analysis of CYP1A1 protein expression in a concentration-response. After co-treatment with TCDD (1 nM) and RSV (at different concentrations as shown above) for 24 hours, (C) mRNA expression of CYP1A1 was detected by RT-PCR. (D) Protein expression of CYP1A1 was detected by Western blot.

In another of our studies we have demonstrated that there was a high level of AhR expression in AGS cells [24]. To determine whether AhR pathway can be activated in this cell line, we examined the expression of Cytochrome P-450 1A1 (CYP1A1), a classic target gene of AhR pathway, following AhR agonist TCDD exposure. RT-PCR and western blot analysis showed that both CYP1A1 mRNA (Fig. 1A) and protein (Fig. 1B) expression in AGS cells were increased in a dose-dependent manner following TCDD treatment. To determine whether this TCDD-induced CYP1A1 expression is AhR-dependent, AhR antagonist resveratrol (RSV) was used to block AhR pathway. As shown in Fig. 1C and 1D, RSV could reverse TCDD-induced CYP1A1 expression in a dose-dependent manner. These data demonstrated that AhR pathway can be activated in AGS cells by TCDD.

### AhR pathway activation in AGS cells induce MMP-9 expression

**Figure 2:**
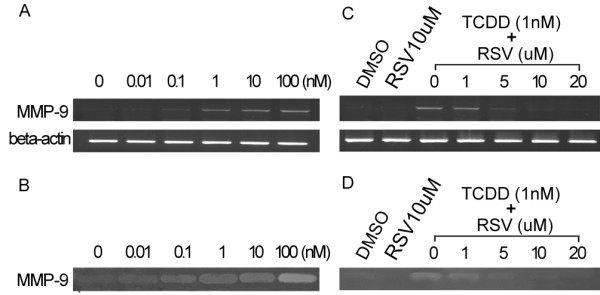
**AhR agonist TCDD and AhR antagonist RSV regulated MMP-9 expression and activity in AGS cells**. After different concentrations (as shown above) of TCDD treatment for 24 hours, (A) RT-PCR analysis of MMP-9 mRNA expression in a concentration-response. (B) Gelatin zymography analysis of MMP-9 protein activity in a concentration-response. After co-treatment with TCDD (1 nM) and RSV (at different concentrations as shown above) for 24 hours, (C) mRNA expression of MMP-9 was detected by RT-PCR. (D) Protein activity of MMP-9 was detected by gelatin zymography.

Previous studies have shown that in several cancer cells, AhR agonist TCDD exposure activates MMP-9 gene expression [18,19]. In order to determine whether TCDD treatment of AGS cells results in an induction of MMP-9 expression, we detected MMP-9 mRNA using RT-PCR following TCDD exposure. As shown in fig. 2A, MMP-9 mRNA expression was induced in a dose-dependent manner following TCDD treatment. To examine the role of the AhR pathway in mediating TCDD-induced MMP-9 expression in AGS cells, cultures were co-treated with TCDD and the AhR antagonist resveratrol. Co-treatment with resveratrol abolished TCDD-induced MMP-9 expression (fig. 2C) demonstrated that TCDD-activation of MMP-9 expression is dependent on the AhR pathway. Above data indicate that AhR pathway activation in AGS cells induces MMP-9 mRNA expression. In most cell types, the gene transcription of MMP-9 is inducible, and after translation the enzyme is immediately secreted and activated through the cysteine-switch mechanism [25]. To determine whether these changes in gene expression following AhR pathway activation result in changes in MMP-9 enzymatic activity, we performed gelatin zymography. The results showed that MMP-9 activity in media is gradually increased with the concentration increase of TCDD in media (fig. 2B) and these changes could be reversed by resveratrol treatment in a dose-dependent manner (fig. 2D). These data demonstrate that AhR pathway activation-induced increase in MMP-9 expression correlates with an increase in MMP-9 activity.

### AhR pathway activation enhances AGS cells migration and invasion

**Figure 3:**
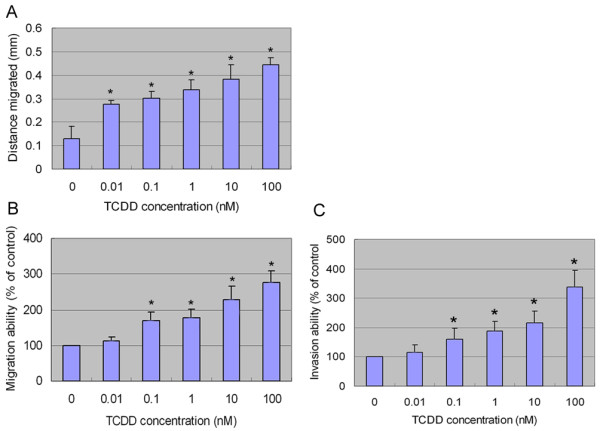
**The effect of AhR agonist TCDD on AGS cells migration and invasion**. (A) Wound healing migration assay. (B) Transwell migration assay. (C) Transwell invasion assay.

MMP-9 is one of the type IV collagenase/gelatinases that can degrade ECM components. MMP-9 is widely associated with tumor invasion and metastasis [17]. Studies have demonstrated a significant correlation between MMP-9 expression and invasiveness and lymph node metastasis of gastric carcinomas [26-28]. To examine whether AhR activation-induced MMP-9 expression and activity in AGS cells results in increased migration and invasion, we performed wound healing migration assay and transwell migration and invasion assay. After TCDD treatment, the migration distance of AGS cells was significantly increased with a dose-dependent manner when compared with control cells (P < 0.01) (fig. 3A). Transwell results showed a significant increase of migration (fig. 3B) and invasion (fig. 3C) abilities of AGS cells when the cells were incubated with TCDD in concentrations from 0.1 nmol/L (P < 0.01) to 100 nmol/l (p < 0.01). no significant effect on the migration and invasion was found at TCDD concentration < 0.1 nmol/l (p > 0.05). These results demonstrated that AhR pathway activation enhances gastric cancer AGS cells migration and invasion. Degradation of ECM and basement membrane is an essential step in tumor invasion and metastasis. It involves the action of matrix metalloproteinases (MMPs). Our study demonstrated that AhR pathway activation could induce MMP-9 expression and enzymatic activity, and promote AGS cells migration and invasion. Other studies indicated that AhR pathway activation could induce a variety of MMPs expression [19,29]. AhR pathway activation enhances gastric cancer AGS cells migration and invasion may be also due to other MMPs expression besides MMP-9 expression.

### c-Jun mediated MMP-9 expression induced by AhR pathway activation

**Figure 4:**
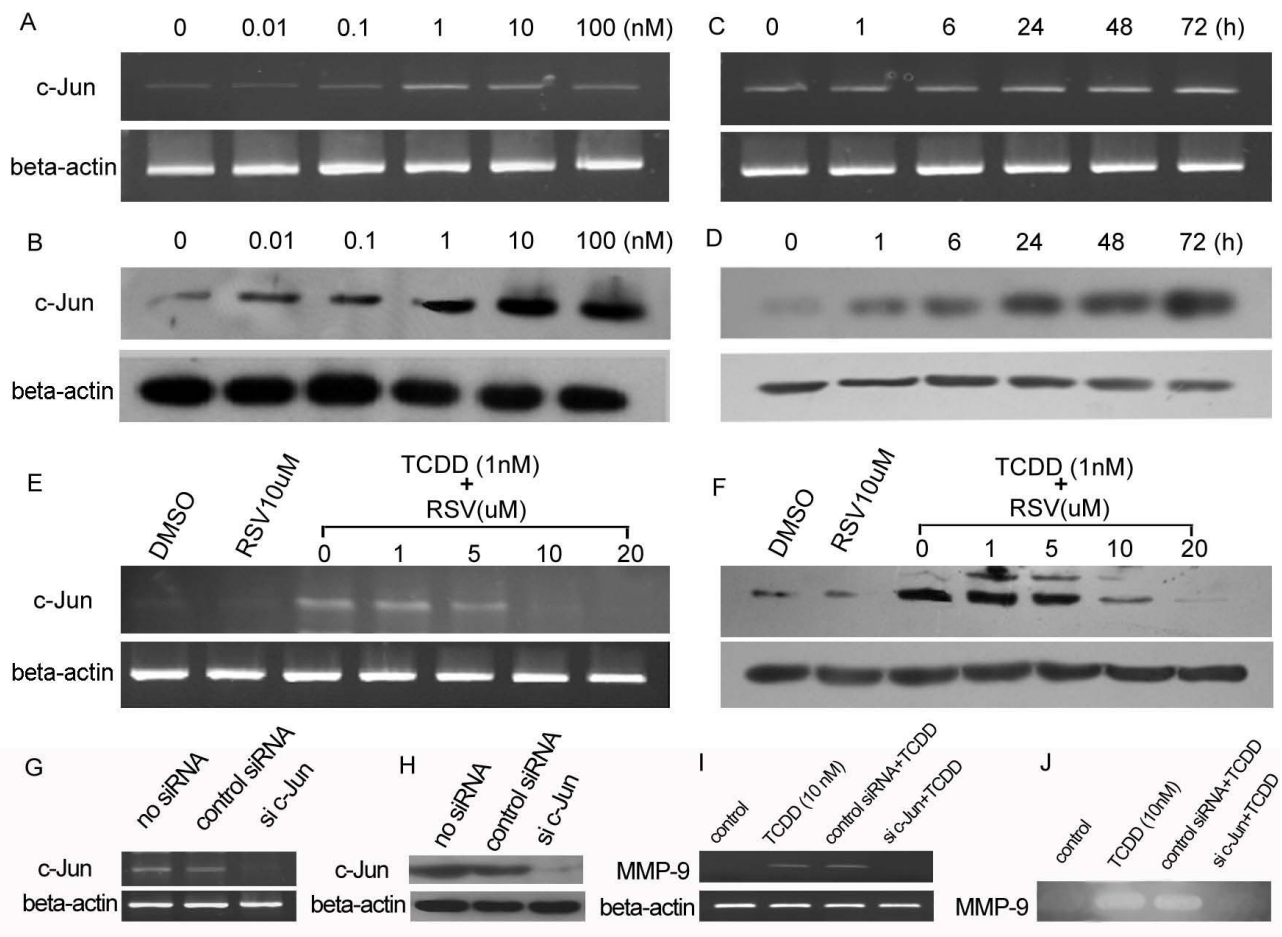
**c-Jun mediates TCDD-induced MMP-9 expression and activity**. (A) TCDD induces c-Jun mRNA expression in a dose-dependent manner. (B) TCDD induces c-Jun protein expression in a dose-dependent manner. (C) TCDD induces c-Jun mRNA expression in a time-dependent manner. (D) TCDD induces c-Jun protein expression in a time-dependent manner. (E) RSV reverses TCDD-induced c-Jun mRNA expression. (F) RSV reverses TCDD-induced c-Jun protein expression. (G) c-Jun siRNA silences c-Jun mRNA expression. (H) c-Jun siRNA silences c-Jun protein expression. (I) c-Jun siRNA inhibits TCDD-induced MMP-9 mRNA expression. (J) c-Jun siRNA inhibits TCDD-induced MMP-9 activity increase.

The mechanism of TCDD-induced changes in MMP-9 expression is not entirely clear. Recent studies reported that TCDD can induce c-Jun expression [30,31] and c-Jun is a target gene of AhR pathway [8]; the promoter sequence in the 5'-flanking region of human MMP-9 gene contains c-Jun binding sites [16] and c-Jun can induced MMP-9 expression [32]; the transcription activity of c-Jun is significant enhanced in gastric cancer [33]. Basing on these study results, we proposed a hypothesis that TCDD-induced MMP-9 expression in AGS cells is mediated by c-Jun. To demonstrate this hypothesis, we first detected the c-Jun expression in AGS cells following TCDD treatment, and found that both c-Jun mRNA (fig. 4A and 4C) and protein (fig. 4B and 4D) expression in AGS cells were increased in a dose (fig. 4A and 4B) and time (fig. 4C and 4D) dependent manner following TCDD treatment, and this TCDD-induced c-Jun expression could be reserved by AhR antagonist resveratrol (fig. 4E and 4F). Above data demonstrated that c-Jun is a target gene of AhR pathway in AGS cells. To further demonstrate that c-Jun can mediate TCDD-induced MMP-9 expression, siRNA transfection was performed to silence c-Jun mRNA in AGS cells. The results demonstrated that c-Jun siRNA (final concentration 50 nmol/L) transfection almost completely abolished c-Jun expression in AGS cells both in mRNA level (fig. 4G) and in protein level (fig. 4H). Therefore, after c-Jun siRNA transfection for 48 hours, AGS cells were treated with TCDD at concentration 10 nmol/L for 24 hours, cell pellet and culture media were collected, and MMP-9 mRNA expression and activity were measured. As shown in fig. 4I and 4J, MMP-9 mRNA expression and activity in untreated control AGS cells were very weak; After TCDD (10 nmol/L) treatment, MMP-9 mRNA expression and activity were significant increased; This TCDD-induced MMP-9 expression and activity increase could be abolished by c-Jun siRNA transfection and not be influenced by control siRNA transfection. Above data demonstrated that c-Jun mediated MMP-9 expression induced by AhR pathway activation in AGS cells.

## Conclusion

In conclusion, the present study demonstrate that AhR pathway can be activated in gastric cancer AGS cells and AhR pathway activation induces MMP-9 expression and activity which ultimately contributes to AGS migration and invasion in vitro. Furthermore, our data show that this AhR activation-induced MMP-9 expression is mediated by c-Jun. Because degradation of ECM and basement membrane is an essential step in tumor invasion and metastasis, our results provide insight into the mechanism and function of the AhR pathway and its impact on these processes during gastric cancer progression.

## Methods

### Cell culture

Gastric cancer cell line AGS was obtained from the American Type Culture Collection (ATCC, Rockville, MD), and was maintained in RPMI 1640 medium (Hyclone) supplemented with 2 mM glutamine, 10% fetal bovine serum (Hyclone), 100 units/ml of penicillin, and 100 μg/ml of gentamycin. Cellular environment was maintained at 5% CO_2_ and 37°C. Cells were harvested from the exponential growth phase and total RNA and protein were prepared as described below.

### Treatment of cells

TCDD and Resveratrol were purchased from Sigma Chemical Company (Bellefonte, PA, USA). After incubation for 24 hours, the cells were treated with TCDD at different concentrations (0, 0.01, 0.1, 1, 10, 100 nM) or TCDD (1 nM) plus Resveratrol (0, 1, 5, 10, 20 μM) for 24 hours, or treated with TCDD (1 nM) for different time (0, 1, 6, 24, 48, 72 hours) respectively. All drugs were dissolved in dimethyl sulphoxide (DMSO). Control cells received 0.1% DMSO only.

### Small interfering RNA synthesis

Small interfering RNAs (siRNAs) were synthesized and high-performance purified (Ribo Biotechnology, Guangzhou). c-Jun siRNAs (sense, CCAAGAACGUGACAGAUGA-dTdT; antisense, UCAUCUGUCACGUUCUUGG-dTdT) and control siRNAs (sense, AGGAGAUAUUUCGAGGCUU-dTdT; antisense, AAGCUCGAAAUAUCUCCU-dTdT), bearing no homology with any known human genes, were dissolved in RNase-free ddH_2_O to a final concentration of 20 μmol/L.

### Small interfering RNA transfection

Cells (5 × 10^5^) were plated onto wells of six-well cell culture plates and allowed to adhere for 24 hours. Four microliters Lipofectamine2000 (Invitrogen) per well were added to serum-free Opti MEM (Invitrogen) for a final complexing volume of 250 μl, gently mixed, and incubated at room temperature for 5 minutes. Five microliters of siRNA solution per well were diluted with 250 μl serum-free Opti MEM. Mix the diluted siRNA solution and the diluted Lipofectamine2000 gently, and incubate at room temperature for 30 minutes. The Lipofectamine2000/siRNA complex was added into the wells containing 1500 μl RPMI 1640 with 10% FBS and incubated in normal cell culture conditions. All of the assays were performed after 48 hours.

### RNA isolation and RT-PCR

Total RNA of cells was extracted using the Quiagen RNeasy Mini Kit (Quiagen) according to the manufacturer's instructions. cDNA was synthesized with 1 μg total RNA using reverse transcriptase, ReverTra AceTM (Toyobo Co, Osaka, Japan) under following condition: 30°C for 10 min, 42°C for 20 min, 99°C for 5 min, and 4°C for 5 min. PCR of cDNA was carried out in a reaction mixture (30 μl) containing 2 μl of template cDNA, 2.5 mM MgCl_2_, 200 μM dNTPs, 0.3 μM primer 1 and 2, and 1 unit of Taq DNA polymerase (New England Biolabs). Amplification was performed using the following condition: 94°C for 5 min, followed by 25–32 cycles (denature for 45 s at 94°C, anneal for 30 s, and extend for 30 s at 72°C), and then 72°C for 7 min. The details of primers, annealing temperature, amplification cycles, and PCR product size for each gene are listed in Table 1. The PCR products were electrophoresed on 1.5% agarose gel, stained with ethidium bromide, and visualized with an ultra-violet (UV) transilluminator. Band intensities in RT-PCR were quantified using Quantity One imaging analysis software. Band intensities of CYP 1A1, MMP-9 and c-Jun mRNA were normalized with corresponding band intensities of beta-actin. Data was reported as mean ± SD.

**Table 1:** Primer sequences and PCR amplification conditions

Gene	Primers (5'→3')	Annealing temperature (°C)	Cycles	Product size(bp)
CYP1A1	**S: **CCATGTCGGCCACGGAGTT	59	32	174
	**A: **ACAGTGCCAGGTGCGGGTT			
MMP-9	**S: **CAACATCACCTATTGGATCC	52.5	37	480
	**A: **CGGGTGTAGAGTCTCTCGCT			
c-Jun	**S: **TCAGACAGTGCCCGAGAT	55.2	30	292
	**A: **CTGCGTTAGCATGAGTTGG			
Beta-actin	**S: **CTCGCTGTCCACCTTCCA	52	30	256
	**A: **GCTGTCACCTTCACCGTTC			

Abbreviations: S, sense primer; A, antisense primer

### Western blot analysis

Cell pellets were homogenized in a lysis buffer containing 20 mM Hepes, 1 mM EGTA, 50 mM β-glycerophosphate, 2 mM Sodium orthovanadate, 10% Glycerol, 1% Triton X-100, 1 mM DTT, and 1× Protease Inhibitor Cocktail (Roche, Mannheim, Germany). Lysate was centrifuged at 13000 rpm and 4 ^o^C for 10 minutes. The supernatant was the total cell lysate. Protein concentration was measured using the BCA protein assay kit (Pierce Chemical Co., Rockford, IL). Thirty micrograms of protein was loaded per lane, separated by 10% SDS-polyacrylamide gel electrophoresis, and transferred onto equilibrated polyvinylidene difluoride membrane by electroblotting. Membranes were blocked with TBS-T buffer containing 5% (w/v) nonfat dry milk. AhR, CYP1A1, c-Jun and beta-actin were detected for 2 hours using antibodies against AhR (SC-5579, Santa Cruz Biotechnology, working dilution 1:150), CYP 1A1 (AB1258, Chemicon International, working dilution 1: 500), c-Jun (SC-1694, Santa Cruz Biotechnology, working dilution 1:200) and beta-actin (#4970, Cell Signaling Technology, working dilution 1: 1000). After secondary antibody incubation (working dilution 1: 2000), enhanced chemiluminescence (Pierce Biotechnology, Inc.) was determined by exposure to x-ray film. Band intensities in Western blot were quantified using Quantity One imaging analysis software. Band intensities of CYP 1A1 and c-Jun were normalized with corresponding band intensities of beta-actin. Data was reported as mean ± SD.

### Gelatin zymography

Media from TCDD treated cultures was electrophoresed on SDS-PAGE where the gel contains 0.1% (w/v) gelatin. The gel was incubated overnight in a Zn^2+^ and Ca^2+^ containing buffer to allow the MMPs to regain their proper structure and degrade the gelatin in the gel. Clear white bands on the Coomassie stained gel are indicative of MMP activity. Band intensities were quantified using Quantity One imaging analysis software.

### Wound healing migration assay

For the measurement of cell migration during wound healing, AGS cells were seeded in individual wells of a 6-well culture plate. When the cells reached a confluent state, cell layers were wounded with a plastic micropipette tip having a large orifice. The medium and debris were aspirated away and replaced by 2.5 ml of fresh serum-free medium which contained different concentrations of TCDD (0, 0.01, 0.1, 1, 10, 100 nM). Cells were photographed every 12 h after wounding by phase contrast microscopy. For evaluation of "wound closure," five randomly selected points along each wound were marked, and the horizontal distance of migrating cells from the initial wound was measured. Data was reported as mean ± SD.

### Transwell migration and invasion assay

Treated or untreated control cells were seeded in triplicate into the upper chamber of a Transwell insert (Corning, New York, NY, USA) in serum-free medium at a density of 1 × 10^5^ per well. For migration assays, medium containing 5% serum was placed in the lower chamber to act as a chemoattractant, and cells were further incubated for 18 h. Nonmigratory cells were removed from the upper chamber by scraping, and the cells remaining on the lower surface of the insert were stained using 0.1% crystal violet for 15 minutes. Cells were counted under a microscope. Invasion assays were done as for the migration assays described above, except inserts were precoated with the extracellular matrix (ECM) substitute Matrigel (BD Biosciences) and incubated over a 24-h period. Each clone was plated in triplicate in each experiment and each experiment was repeated at least thrice. Cell migration (or invasion) ability = (the cell number of treated group/the cell number of control group) × 100%.

## Authors' contributions

PTL and CJ contributed equally to this work; PTL and CJ performed research and wrote the paper. MW organized the figures and analyzed data. SX participated in the study design. CMH designed research and supervised the writing and organization process. All authors read and approved the final manuscript.
